# Circle plus Partial Helical Scan Scheme for a Flat Panel Detector-Based Cone Beam Breast X-Ray CT

**DOI:** 10.1155/2009/637867

**Published:** 2009-12-31

**Authors:** Dong Yang, Ruola Ning, Weixing Cai

**Affiliations:** Department of Imaging Sciences, University of Rochester Medical Center, 601 Elmwood Avenue Rochester, NY 14642, USA

## Abstract

Flat panel detector-based cone beam breast CT (CBBCT) can provide 3D image of the scanned breast with 3D isotropic spatial resolution, overcoming the disadvantage of the structure superimposition associated with X-ray projection mammography. It is very difficult for Mammography to detect a small carcinoma (a few millimeters in size) when the tumor is occult or in dense breast. CBBCT featured with circular scan might be the most desirable mode in breast imaging due to its simple geometrical configuration and potential applications in functional imaging. An inherited large cone angle in CBBCT, however, will yield artifacts in the reconstruction images when only a single circular scan is employed. These artifacts usually manifest themselves as density drop and object geometrical distortion that are more noticeable in the reconstructed image areas that are further away from the circular scanning plane. In order to combat this drawback, a circle plus partial helical scan scheme is proposed. An exact circle plus straight line scan scheme is also conducted in computer simulation for the purpose of comparison. Computer simulations using a numerical breast phantom demonstrated the practical feasibility of this new scheme and correction to those artifacts to a certain degree.

## 1. Introduction

Breast cancer imaging has improved over the last decade with higher and more uniform quality standards for mammography as well as through the increasing use of sonography and magnetic resonance imaging as the adjunct tools. Mammography is still the only screening tool to detect breast cancer for asymptomatic women. Due to the limitations associated with the aforementioned techniques, such as imaging of the overlapping structure with mammography, technician dependent lack of ability to detect calcifications with ultrasound, and low specificity and/or poor detection of the tiny calcium deposits with MRI, there remains an endeavor to explore new ways to better detect breast cancer. 

Recently one of the most exciting ways is cone beam breast CT (CBBCT) technology [1–4]. It is based on a flat panel detector, and with only one circular rotation or some other scanning path, it can provide the three-dimensional density distribution of the breast, thus greatly eliminating the imaging problem of the structure overlapping seen in mammography to enhance the contrast resolution. It has been shown that the average glandular doses of CBBCT is equivalent to mammography [5, 6]; so this technology might have the potential to replace mammography for breast cancer screening and diagnosis. 

Among all CBBCT technologies, FDK [7, 8] algorithm-based circular scan scheme possesses the following advantages: a stable and simple mechanical configuration, motion artifacts reduction, computation efficiency, and so forth. However, since a single circular source trajectory does not satisfy the data sufficient condition [9], the FDK algorithm will unavoidably induce some artifacts such as an intensity drop along the rotation axis and object geometric distortion in the area further away from the circular scanning plane when cone angle becomes large. In order to overcome these cone beam artifacts, we propose the circle plus partial helical (CH) scan scheme based on the idea that by partially filling the object support in the Radon domain (i.e., the well-known torus in 3D Radon domain) where the circular scan does not touch through the additional scanning trajectory (such as a partial helical orbit), we can acquire more information than from just a single circular scan. The idea behind the partial helical scan is to improve the image quality by correcting the aforementioned artifacts to a certain degree while not exposing the patient with too much radiation exposure. In order to maintain computation efficiency, a filtered backprojection (FBP) method is employed for the reconstruction part associated with partial helical scan. 

Recently, Katsevich and Kapralov [10] proposed a circle plus general curve scan algorithm for exact reconstruction, which is also of FBP type; moreover, it is an exact shift-invariant algorithm and very computationally efficient. The requirements for this additional scan are that, first, this additional general curve has to be a piecewise smooth curve (i.e., a straight line or helix); second, during this additional scan the circle trajectory must find its projection on the detector as it is seen from the X-ray source. General CT scanner and C-arm can easily meet this requirements and exact ROI reconstruction can be achieved by employing this algorithm. In case of CBBCT prototype, however, it is better to keep the X-ray collimation fixed (i.e., half cone illumination) during additional noncircular scan to reduce the system complexity since the scanner possesses a half cone geometry covering the whole detector. So the second requirement with respect to the aforementioned Katsevich algorithm is hard to meet. Based on this special geometric requirement of CBBCT, the proposed partial helical scan part will be reconstructed using a shift-variant filtered-backprojection [11]. When variable size collimation is available, Katsevich type reconstruction can be conducted along a straight line scan in numerical simulation. The hybrid reconstruction method is adopted for both cases. For the proposed CH scheme, the reconstruction is composed of three parts: FDK term for circle [7], Hui's term for circle [12], and a shift-variant FBP term for partial helical scan, whereas for circle plus straight line (CL) scheme, the reconstruction is composed of two terms, circle and straight line reconstructions [13]. Instead of using Hilbert reconstruction for circle part presented by original algorithm, FDK was used due to the better computational efficiency and spatial resolution [14]. Results from both cases are compared and discussed. Overall, computer simulations based on the numerical breast phantom verified that the proposed CH scheme outperforms the FDK-based single circular scan scheme.

## 2. Methods and Materials

### 2.1. Data Acquisition Analysis in Terms of Radon Domain

It is well known that a single circular cone beam scan does not provide complete information for an exact reconstruction. This can be appreciated by the 3-D Radon transform of the object function$\;f(\vec{r})$, which is mathematically shown as

(1) $$Rf\left(\rho \vec{\varepsilon }\right)=\int \;f\left(\vec{r}\right)\cdot \delta \left(\vec{r}\cdot \vec{\varepsilon }-\rho \right)d\vec{r}.$$

**Figure 1:**
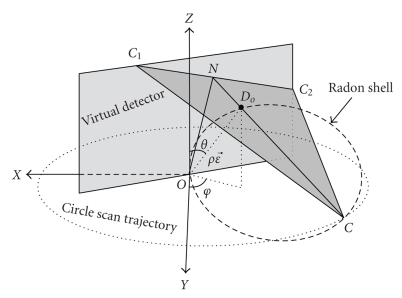
Illustration of 3-D Radon transform and Radon shell in object space.

**Figure 2:**
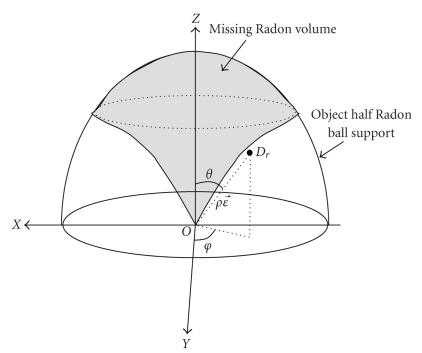
Illustration of the radon point in the radon domain within object Radon support.

The equation above represents a 3-D Radon transform of $f(\vec{r})$ along the plane defined by $\vec{r}\cdot \vec{\varepsilon }=\rho$. One of the properties of 3D Radon transform is that an object with a spherical support in object space has the same size of spherical support in Radon space. In cone beam projection, the distance between the X-ray source and the rotation center is the diameter that determines a spherical Radon shell where the points on this Radon shell are Radon points in Radon space. Their values are represented by the integral of the plane that is defined by $\vec{r}\cdot \vec{\varepsilon }=\rho$ in the object space. Figure 1 illustrates the 3-D Radon transform and concept of the spherical Radon shell. *XOY* defines a scanning plane, and the point *C* represents one X-ray source on the circle scan trajectory; *O* is the rotation center; *OC* is the diameter by which a Radon shell is defined; *D*_*o*_ is a point on the Radon shell; *N* is a point where the line *CD*_*o*_ intersects the virtual detector. *C*_1_ *C*_2_ is a line that crosses the point *N* and is perpendicular to the line *ON*, *CC*_1_ *C*_2_ defines a plane (i.e., Radon plane) where its normal is $\vec{\varepsilon }$, and the distance from the rotation center *O* to this plane which is also the length of the line *OD*_*o*_ is *ρ*. The corresponding Radon point *D*_*r*_ in Radon domain that is defined by the Radon plane *CC*_1_ *C*_2_ in the object space is illustrated in Figure 2. During a circular scan, this spherical Radon shell sweeps around the rotation axis (*Z* axis) to constitute a torus in a 3-D Radon domain. In the CBBCT scanning geometry, the aforementioned Radon shell becomes a half Radon shell on the scanning plane; so only a half Radon ball is shown.

The light gray volume inside the half Radon ball support is what is called the missing volume, meaning that no Radon points in this volume can be acquired through circular scan. In the spherical coordinates, this missing Radon volume is expressed as *ρ* > *OC* · sin*θ*. We can make two claims by observing this missing Radon volume. (1) When the sampling rate is fixed, more Radon points are needed to fill this missing volume in the part further away from the scanning plane than in the part closer to the scanning plane. This actually indicates that the reconstruction based on the circular scan has more artifacts in the reconstructed slices that further away from the scanning plane than those closer to the scanning plane. (2) The ratio of the radius of the object support and the diameter of the spherical Radon shell determines the size of the missed Radon data volume, which results in the reconstructed object that is closer to or farther away from the exact reconstruction. With a fixed diameter (i.e., the distance between the X-ray source and the rotation center) of the Radon shell, it is evident that the smaller the breast, the better the reconstructed image; the bigger the breast, the worse the reconstructed image in terms of artifacts.

According to Chen and Ning [1], when the scanning half cone angle spanned by the breast is within 8 degrees, the circular-based modified FDK (MFDK) [12] (which is the addition of first two terms in CH scheme) still provides clinically acceptable reconstructed images. However, as the half cone angle gets bigger than 8 degrees, artifacts such as density drop and geometrical distortion are more noticeable in the reconstructed images based on a single circular scan. An additional scanning trajectory should be added to fill the missing Radon data volume in order to produce clinically acceptable images. Based on the claims made in the previous paragraphs, the filling of the missing Radon volume probably does not need to be complete. In other words, only part of the missing Radon volume need to be filled so as to correct to a certain degree of the artifacts associated with a single circle scan. Also note that in practical CBBCT imaging this missed volume is actually a small portion in the half ball Radon support of the object. The sampling rate of Radon data within this volume does not need to be as high as it does in the volume acquired through a circular scan. These realizations can help us give the patient not too much extra X-ray exposure by introducing an auxiliary scanning trajectory and improve image quality to a certain degree as well. 

There are a couple of proposed “circle plus” trajectories [11, 13, 15–18]. Due to the special geometrical configuration of the CBBCT, the circle plus arc is not applicable; however the CL seems to be applicable. For ease of operation and in order to avoid unnecessary extra X-ray exposure, the X-ray half cone collimation associated with the circle scan must be kept for line scan trajectory. If the line-scan trajectory is described as *ϕ*_*L*_(*l*) = (0, *m*, *l*), where *m* is a constant in the *Y*-axis, and *l* is a variable along the *Z*-*axis*, representing the line scan X-ray shot position, based on the illustration from Figure 1, we can see that only a half Radon shell associated with each X-ray position during line scan can be defined and it is tangential to the *XOZ* plane. In Figure 2, the filling of the Radon data from this line-scan can only be added in half of the missing Radon volume separated by the *XOZ* plane. Since the missing Radon volume is symmetrical around the rotation axis (*Z* axis), then this unsymmetrical filling of the Radon data in terms of projection angle in the missing Radon volume may not achieve the best reconstruction result. By taking advantage of the circular scanning feature of CBBCT, one way to combat this unsymmetrical filling is to lower down the X-ray tube and detector while simultaneously rotating them around the breast to achieve an approximate symmetrical filling of the Radon data in the missing Radon volume. It is like the helical scan but with sparse X-ray shots at positions described as *ϕ*_HL_(*β*_*i*_, *l*_*i*_) = (*D*cos *β*_*i*_, *D*sin*β*_*i*_, *l*_*i*_), where *D* is the distance from the X-ray tube to the rotation center, and *l*_*i*_ is the position along the *Z* axis, and can be described as *l*_*i*_ = *l*_0_ + (*i* − 1)Δ*l*, where *l*_0_  is the starting position along the *Z* axis for this partial helical scan, *i* is index of X-ray shot, Δ*l* is the line increment along the *Z* axis, and *β* is the projection angle, and also can be described as *β*_*i*_ = (*i* − 1)Δ*β*, where Δ*β* is the projection angle increment in the unit of radians. The Radon data acquired through this scanning trajectory can fill the part of this missing Radon volume. Thus the result is not an exact reconstruction. The key point here is to introduce the additional scanning trajectory so as to correct to a certain degree the reconstruction artifacts associated with a single circular scan.

For comparison, a CL scanning is also conducted in numerical simulation based on Katsevich's concept under the less restrictive conditions. During the line scanning, the detector is always fixed at the position where circle scan is conducted, and the X-ray collimation size varied to make sure that X-ray illumination always covers the whole detector as it moves along the line trajectory. In this way, the missing radon volume is filled completely and an exact reconstruction can be achieved through CL scan.

### 2.2. Scan Design for the Partial Helical Scan and Straight Line Scan Trajectory

Based on the geometric parameters of current CBBCT, we designed a new scan scheme. The position of the X-ray source is at *z* = 0 cm during the circular scan. After the circular scan, the X-ray source and detector lower down simultaneously while they are still rotating. When the X-ray source gets to the point where *z* = *l*_0_ (we will talk later how we choose the *l*_0_), it starts to shoot at positions described as *ϕ*_HL_(*β*_*i*_, *l*_*i*_) = (*D*cos *β*_*i*_, *D*sin*β*_*i*_, *l*_*i*_) (*i* = 0 to *n*), and the X-ray source maintains the same half cone illumination for each shooting as it is with circular scan; so part of the breast in each helical shot between *z* = 0 and *z* = *l_i_* (*i* = 0 to *n*, *n* is total number of X-ray shots during partial helical scan) can avoid being exposed by the X-ray. 

The projection angles associated with partial helical scans are uniformly distributed within 2*π* range. There are32 and 64X-ray shots during partial helical scans that uniformly cover the angular range of 2*π*, and the movement in the *Z* direction is from 49 mm to 121 mm with the increment interval of 2.34 and 1.15 mm based on the size of the simulated breast phantom. The reason we chose the starting position at *Z* = 49 mm for partial helical scan is because we found that based on our simulated scanning geometrical parameter the attenuation coefficient drop in the regular circular scan started approximately at *Z* = 49 mm.

Some of the Radon data points acquired from this additional scanning trajectory still can be acquired through a circular scan. This is what is called redundant sampling points in the Radon domain and can be efficiently eliminated by a window function. The geometric setup of the collimation during the partial helical scan is maintained as it is with the circle scan, that is, the half cone illumination geometry. This can avoid the redundant sampling in the missing volume in the Radon domain within the X-ray shots in a helical trajectory. Since the collimation during partial helical scan unavoidably encounters the longitudinal truncation, a geometric dependent truncation window function has to be used to handle this case to remove the incorrect Radon data.

**Figure 3:**
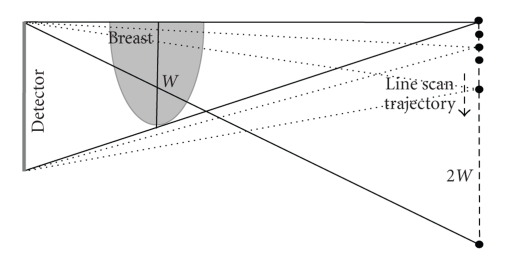
Illustration of the straight line scan to achieve exact reconstruction.

In line scan case, as Figure 3 shows, the virtual detector length along the circular rotation axis is *W*. Those little black dots represent the X-ray source at different positions in line scan. During the line scan, the detector is fixed and the collimation of the X-ray is adaptively changed to cover the whole detector, and the length of this scanning line is 2*W*. The circle trajectory can always be projected onto the detector as it is seen from the X-ray source during the straight line scan, thus enabling us to use Katsevich's algorithm to do the reconstruction for this line scan part.

### 2.3. FBP Reconstruction Algorithm Associated with Different Scan Schemes

#### 2.3.1. Algorithm for CH Scan Scheme

**Figure 4:**
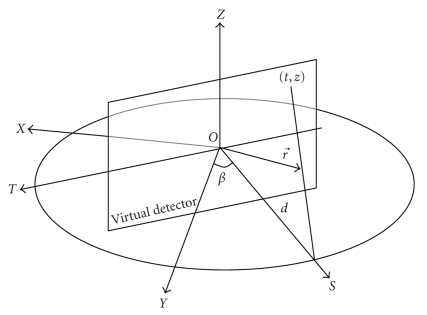
The geometric illustration of a circular scan.

Composite reconstruction framework is probably the most preferable algorithm for the CBBCT. The reconstructed object is$\;f(\vec{r})$ and can be mathematically described by the following equation:

(2) $$f\left(\vec{r}\right)=f_{\text{cir}}\left(\vec{r}\right)+f_{\text{Hui}}\left(\vec{r}\right)+f_{\text{HL}}\left(\vec{r}\right),$$

where $f_{\text{cir}}(\vec{r})$ is the reconstructed object from a single circular scan; $f_{\text{Hui}}(\vec{r})$ is the reconstructed object from Hui's term based on a single circular scan; $f_{\text{HL}}(\vec{r})$ is the reconstructed object from a partial helical scan;

Figure 4 describes circular scan geometry.

The mathematic equation of $f_{\text{cir}}(\vec{r})$ and $f_{\text{Hui}}(\vec{r})$ can be expressed by (3) and (5), respectively, as follows.

(i) FDK algorithm:

(3) $$f_{\text{cir}}\left(\vec{r}\right)=\frac{1}{4{\;\pi }^{2}}\oint \frac{d^{2}}{{\left(d+\vec{r}\cdot \vec{s}\right)}^{2}}P_{1}\left(t,z\right)d\beta ,$$

where

(4) $$\begin{array}{c} P_{1}\left(t,z\right)=\int \frac{d}{\sqrt{d^{2}+t^{\prime 2}+z^{2}}}\;P_{\beta }\left(t^{\prime },z\right)h\left(t-t^{\prime }\right)dt^{\prime },\\ t=d\frac{\vec{r}\cdot \vec{T}}{d+\vec{r}\cdot \vec{S}},\;\;z=d\frac{\vec{r}\cdot \vec{Z}}{d+\vec{r}\cdot \vec{S}}, \end{array}$$

where *h*(*t*) is the impulse response of the regularized ramp filter; *P*_*β*_(*t*, *Z*) is the cone beam projection data.

(ii) Hui's term:

(5) $$f_{\text{Hui}}\left(\vec{r}\right)=-\frac{1}{4\pi^{2}}\oint \frac{z}{{\left(d+\vec{r}\cdot \vec{s}\right)}^{2}}P_{2}\left(z\right)\;d\beta ,$$

where

(6) $$\begin{array}{c} P_{2}\left(z\right)=\frac{\partial }{\partial z}\int \frac{d}{\sqrt{d^{2}+t^{2}+z^{2}}}P_{\beta }\left(t,z\right)\;dt,\\ z=d\frac{\vec{r}\cdot \vec{Z}}{d+\vec{r}\cdot \vec{S}}\;. \end{array}$$

Helical scan term:

(7) $$\begin{array}{cc} f_{\text{HL}}\left(\vec{r}\right) & =-\frac{1}{4\pi^{2}\left(d+\vec{r}\cdot \vec{s}\right)}\int_{Z_{0}}^{Z_{n}}dZ\int_{-\pi /2}^{\pi /2}H_{Z_{i}}\left(l,\varphi \right)d\varphi ,\\ H_{Z_{i}}\left(l,\varphi \right) & =\left|\cos\;\;\varphi \right|w_{Z_{i}}\left(l,\varphi \right)w_{tr_{Z_{i}}}\left(l,\varphi \right)\\ & \;\times \left(\frac{2l}{d^{2}}\frac{\partial \sum_{Z_{i}}\left(l,\varphi \right)}{\partial l}+\frac{d^{2}+l^{2}}{d^{2}}\frac{\partial^{2}\sum_{Z_{i}}\left(l,\varphi \right)}{\partial l^{2}}\right),\\ \underset{Z_{i}}{\sum }\left(l,\varphi \right) & =\iint \frac{d}{\sqrt{d^{2}+t^{2}+Z^{2}}}+P_{Z_{i}}\left(t,Z\right)\\ & \;\;\;\times \delta \left(t\sin\;\varphi +Z\cos\;\;\varphi -l\right)dt\;dZ,\\ w_{Z_{i}}\left(l,\varphi \right) & =\{\begin{array}{cc} 1, & 2lZ_{i}\cos\;\;\varphi +Z_{i}^{2}{\cos\;}^{2}\;\varphi -d^{2}\sin^{2}\;\varphi >0,\\ 0, & \text{otherwise,} \end{array}\\ w_{tr_{Z_{i}}}\left(l,\varphi \right) & =\{\begin{array}{cc} 1, & \text{line}\;c_{1}c_{2}\;\text{does}\;\text{not}\;\text{cross}\;\text{the}\;\text{region}\;\text{of}\;\Omega ,\\ 0, & \text{line}\;c_{1}c_{2}\;\text{crosses}\;\text{the}\;\text{region}\;\text{of}\;\Omega . \end{array} \end{array}$$

**Figure 5:**
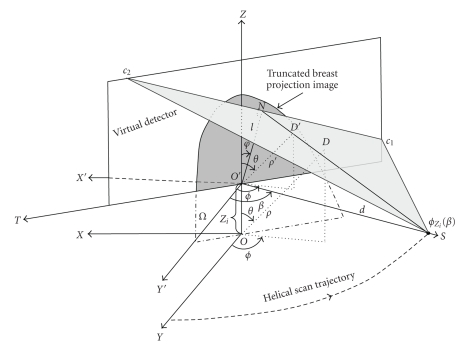
The Geometrical illustration of the same Radon value defined in object coordinates and reconstruction coordinates associated with the partial helical scan.

Based on Figure 5, the reconstruction term for partial helical scan can be formatted as a type of filtered backprojection (FBP) based on the 3-D Radon inversion formula [11]. The mathematic equation of $f_{\text{HL}}(\vec{r})$ is expressed as (7). As was stated in Section 2.1, a redundant window function *w*_*Z*_*i*__(*l*, *φ*) is used to remove Radon points that are acquired through partial helical scan but have already been touched by previous circular scan during the reconstruction. As Figure 5 shows, Radon plane *SC*_1_ *C*_2_ defined in the reconstruction coordinates during partial helical scan corresponds to a Radon point expressed as (*ρ*′, *ϕ*, *θ*) in terms of spherical coordinates. This Radon point must be mapped to the Radon domain defined by the object coordinates expressed as (*ρ*, *ϕ*, *θ*) in order to construct the window function *w*_*Z*_*i*__(*l*, *φ*).  *P*_*Z*_*i*__(*t*, *Z*) is the projection data associated with each X-ray position during partial helical scan.

This helical reconstruction formula is actually similar to what was presented by Hu [11], except that a partial longitudinal truncation window function *w*_*tr*_*Z*_*i*___(*l*, *φ*) is included in this paper. Based on the scanning design, the partial helical scan will unavoidably encounter the longitudinal truncation during the scan. Some Radon points it acquires do not reflect the actual Radon data and should be removed during the back-projection [19]. Window function *w*_*tr*_*Z*_*i*___(*l*, *φ*) is used to achieve this purpose.

#### 2.3.2. Algorithm for CL Scan Scheme

The final reconstruction is composed of two parts, first one is from circular scan, the second one is from straight line scan, and can be mathematically described by the following equation:

(8) $$f\left(\vec{r}\right)=f_{\text{cir}}\left(\vec{r}\right)+f_{\text{line}}\left(\vec{r}\right).$$

**Figure 6:**
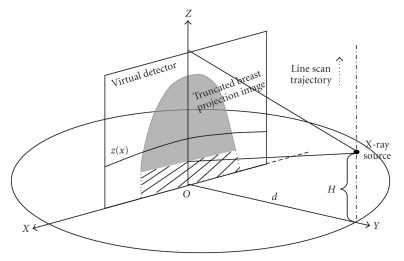
Illustration of straight line scanning.

$f_{\text{cir}}(\vec{r})$ is described by (3), and $f_{\text{line}}(\vec{r})$ will be reconstructed using Katsevich's algorithm. Figure 6 geometrically illustrates the straight line scan. The curve described by *z*(*x*) on the virtual detector is the projection of circle trajectory seen from the current X-ray source. $f_{\;\text{line}}(\vec{r})$ is mathematically described as

(9) $$\begin{array}{cc} f_{\text{line}}\left(\vec{r}\right) & =-\frac{1}{2\pi^{2}}\int \frac{1}{\left|y\left(\vec{l}\right)-\vec{r}\right|}\\ & \;\;\;\;\times \int_{0}^{2\pi }\frac{\partial }{\partial l}P\left(y\left(\vec{l}\right),\Theta \left(\vec{l},\vec{r},\gamma \right)\right)\frac{dy}{\sin\;y}dl. \end{array}$$

The implementation of the $f_{\text{line}}(\vec{r})$ can be referred to [20, 21]. Please note that under the current CBBCT geometry, the curve *z*(*x*) is described mathematically as

(10) $$z\left(x\right)=\frac{H}{2}\left[1-{\left(\frac{x}{d}\right)}^{2}\right].$$

Apparently, this is a parabola with its vertex at (0, *H*/2), where *z* and *x* are the vertical and horizontal coordinates on the detector, and *H* is the distance of X-ray source to the circular scanning plane. The filtering lines (on which the Hilbert filtering are conducted) are determined by the intersection of the flat panel detector with the planes tangent to the curve *z*(*x*). On the detector this line can be described as *z*_*l*_(*x*) = *Kx* + *b*, where *b* > *H*/2. By inserting this line equation into (10), the tangent filtering lines can be described as

(11) $$z_{l}\left(x\right)=\pm \frac{\sqrt{2Hb-H^{2}}}{d}x+b,$$

where *b* is actually the intersection of those lines with the *Z* axis and can be used as an index parameter. Note from (11) that there are two sets of filtering lines that can provide the double coverage of the detector area above the curve *z*(*x*). Hilbert filtering on these two sets of lines should be carefully treated since Hilbert filtering is sensitive to the filtering direction. In the current simulation, contributions from these two sets of filtering lines are added.

## 3. Computer Simulation

### 3.1. Description of the Mathematic Breast Phantom and Scanning Parameter Settings

Computer simulations are carried out on a mathematic breast phantom that was created for this study. This breast phantom is a half-ellipsoid with three half-axes of 8.8, 8.8, and 16 cm, a large phantom, specifically designed to address the artifacts resulting from the single circular scan. The phantom is wrapped by simulated skin with a thickness of 2 mm. Within the simulated skin, the base material is a compound of adipose and glandular tissues (e.g., 50% adipose and 50% glandular). There are three groups of objects inside the breast phantom. Within first two groups are two sets of spheres: one set of carcinoma spheres with diameters of 1, 2, 4, 6, and 8 mm, respectively, located at the positions where *Z* = 10, 70, 130 mm from the chest wall, and one set of glandular spheres with diameters of 1, 2, 4, 6, and 8 mm, respectively, located at the same position as the group of carcinoma spheres. The third group is composed of two low contrast disk-type objects specifically constructed to address the geometrical distortion of the reconstructed objects around the nipple area located at the position where *Z* = 148 mm from the chest wall. The disc length along the *X*-, *Y*-, and *Z -axis* is 10, 10, and 2.5 mm, respectively. The linear attenuation coefficients with respect to skin, base material, carcinoma, glandular, and disk-type object are 0.22, 0.19, 0.23, 0.24, and 0.21, respectively, in unit of 1/cm. The distance between the X-ray source and the rotation center is 650 mm and the detector pixel size is 0.388 mm; the magnification factor is 1.43; the detector size is 661 by 661. The value of the reconstructed images is converted to CT number by using the 0.25 as the linear attenuation coefficient of water. Tables 1 and 2 summarize scanning parameters associated with two auxiliary scan schemes.

### 3.2. Results

#### 3.2.1. Performance with a Different Sampling Interval during Partial Helical Scan

**Figure 7:**
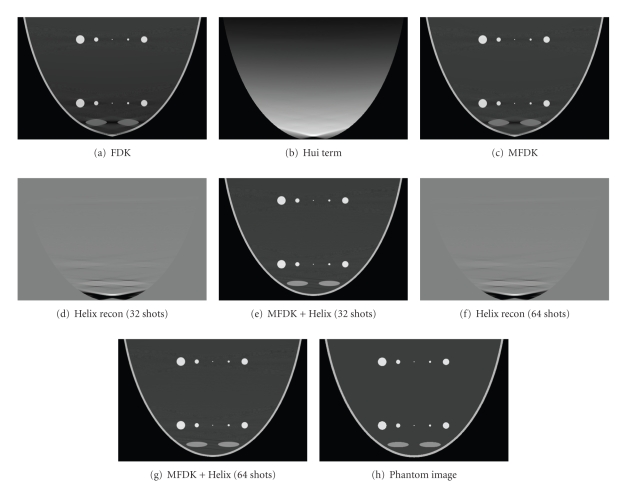
Central sagittal image comparison between MFDK, phantom, and circle plus partial helical term with different sampling intervals. (a) Circular FDK reconstruction; (b) circular Hui's term; (c) MFDK reconstruction (circle FDK + Hui term); (d) partial helical reconstruction (32 X-ray shots during helix scan); (e) MFDK + Helix reconstruction (32 shots for helical scan); (f) partial helical reconstruction (64 X-ray shots during helix scan); (g) MFDK + Helix reconstruction (64 shots for helical scan); (h) phantom image of the same sagittal slice.

The simulation was conducted in several settings as discussed in Section 2.1.Figure 7 illustrates the comparison of the central sagittal images from CH scheme with different sampling intervals in the helical scan and phantom. The angular scanning range is 2*π* within a partial helical scan. The objects at different layers within the breast are simulated tumors with different sizes. The display window is [−300 –100] except Figures 7(b), 7(d), and 7(f).

#### 3.2.2. Performance with a Different Sampling Interval during Straight Line Scan

**Figure 8:**
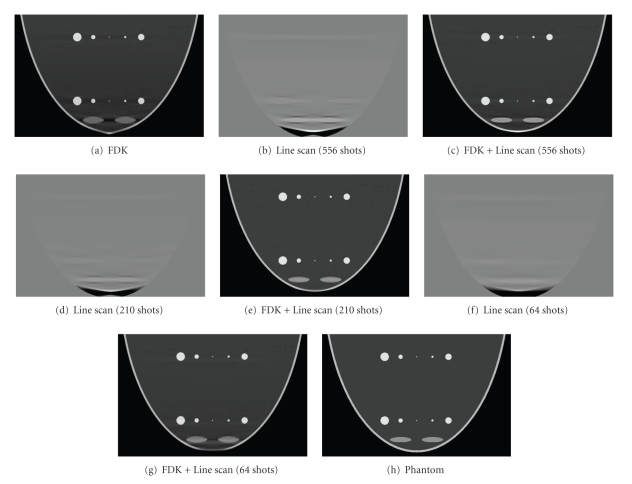
The central sagittal image comparison between phantom and CL scan scheme with different sampling intervals along straight line trajectory. (a) Circular FDK reconstruction; (b) straight line scan reconstruction (556 shots); (c) FDK + Line reconstruction (556 shots for line scan); (d) straight line scan reconstruction (210 shots); (e) FDK + Line reconstruction (210 shots for line scan); (f) straight line scan reconstruction (64 shots); (g) FDK + Line reconstruction (64 shots for line scan); (h) phantom image of the same sagittal slice.

The contribution from straight line scan was reconstructed using Katsevich's algorithm. Figure 8 shows the central sagittal image comparison between phantom and CL scan scheme with different sampling interval along the line scan trajectory. The display window is [−300 –100] except Figures 8(b), 8(d), and 8(f).

#### 3.2.3. Profile Comparison between Phantom, MFDK, CH and CL Scan Schemes

**Figure 9:**
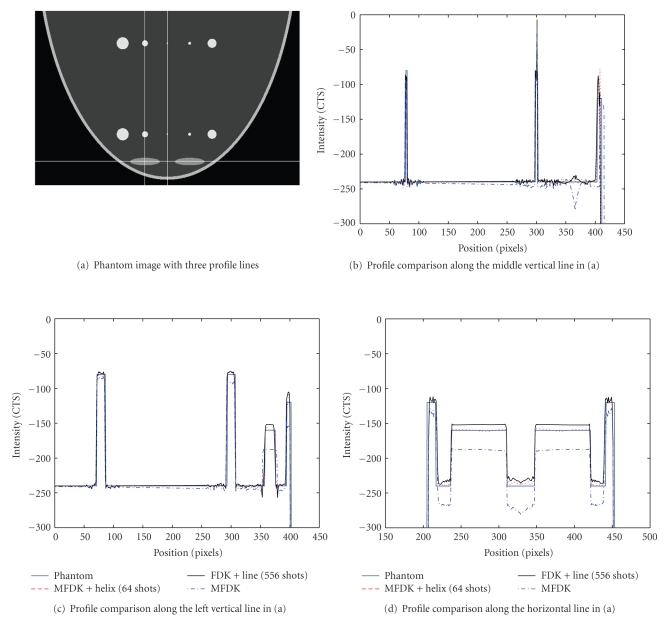
Profile comparison between phantom, MFDK, MFDK plus helical scan, and FDK plus straight line scan schemes. (a) Phantom image with three profile lines; (b) profile comparison along the middle vertical line in (a); (c) profile comparison along the left vertical line in (a); (d) profile comparison along the horizontal line in (a).

Figure 9 shows profile comparison between phantom, MFDK, MFDK plus helical scan, and FDK plus straight line scan schemes. 

#### 3.2.4. Performance Comparison between MFDK, CH, and CL, in Terms of Reconstruction Error

A quantitative measurement of reconstruction error (*RE*) is conducted according to the following formula:

(12) $$RE\left(\text{\%}\right)=\frac{\sum_{i=0}^{N-1}\left|I_{r}\left(i\right)-I_{p}\left(i\right)\right|}{\sum_{i=0}^{N-1}\left|I_{p}\left(i\right)\right|},$$

where *N* is the total pixel number of the central sagittal image; *I*_*r*_(*i*) is the CT number of the *i*th pixel in the reconstructed central sagittal image; *I*_*p*_(*i*) is the CT number of the same pixel in the central sagittal phantom image. Table 3 summarizes the *RE* from different scan schemes.

#### 3.2.5. Performance over Simulated X-Ray Quantum Noise

**Figure 10:**
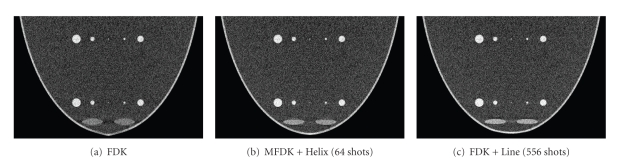
The Central sagittal image comparison between different scanning schemes based on simulated quantum noise in the projection data. (a) Circular FDK; (b) MFDK + Helix reconstruction (64 shots for helical scan); (c) FDK + Line reconstruction (556 shots for line scan).

In order to test the performance of this new scheme over the quantum noise that is commonly encountered in practical CBBCT data acquisition, we generated quantum noise contaminated data. An X-ray with 60 kVp was selected which corresponds to an effective photon fluence of 2.65*10^7^ photons/cm^2^ · mR [22]. The exposure level per projection was set to 4 mR; so the total exposure level for a circular scan is 1200 mR, for CH in which helix scan has 64 points is 1456 mR and for CL in which line scan has 556 points is 3424 mR. Figure 10 shows the central sagittal image from different scanning schemes when projection is contaminated by quantum noise. The display window is [−320 −100].

## 4. Conclusion and Discussion

The new scanning scheme of CH scan works better than a single circular scan in terms of image uniformity and geometrical correctness based on the computer simulations of a mathematic breast phantom and a simulated breast phantom on CBBCT prototype study. Partial helical scan with different sampling intervals showed that the number of X-ray shootings between 32 and 64 could provide acceptable reconstructed images in terms of correction to the intensity drop along the scanning axis and geometrical distortion around the nipple area based on the scanning geometrical parameters and breast size. This is encouraging, since the quality of reconstructed images could improve without too much additional radiation exposure to the patient. Also note that the smaller the sampling interval (the larger the number of projections) in helical scan, the less the streak artifacts in the corrected area. However, these streak artifacts are faintly visible. In practical situation, the image quality should be balanced with the sampling interval in helical scan. This new scanning scheme is not intended to conduct an exact reconstruction. Theoretically, when the missing volume in Radon domain is completely filled and at least as densely sampled as those accessed by circle scan, the combined reconstruction is exact. By sparsely sampling the missing volume through a proposed scanning scheme, it suffices to correct the artifacts occurring in a single circular scan. On the other hand, the new scanning scheme is easy to operate in practice without complicated mechanical modification on the current prototype CBBCT system. 

As was mentioned in Section 2.3.2, an exact FBP type reconstruction was also conducted in numerical breast phantom simulation based on the concept proposed by Katsevich about circle plus general trajectory scanning [10]. Three sampling intervals were simulated in the line scanning reconstruction, the first is one and a half times the size of an actual pixel pitch, the second is four times bigger, and the third is thirteen and three quarters times bigger. Katsevich's algorithm [13] was used for reconstruction. The results shown in Figure 8 indicated that the bigger the sampling interval, the more blur the edges of the reconstructed objects, and when sampling interval was increased to the point that only 64 X-ray shots required to cover the scanning length, some geometrical distortions were still observed in the combined image (Figure 8(g)). In Katsevich's algorithm, the Hilbert filtration is conducted on the differentiated projection data which was approximated by the difference of two adjacent projections divided by sampling interval, and X-ray source corresponding to the Hilbert filtered difference projection data was assumed to be at the position that is in the middle of these two adjacent corresponding X-ray source positions. Since difference of two adjacent projection data can be thought as filtering, so the bigger the sampling interval, the poorer the spatial resolution, and the subtracted data may not correctly reflect the actual projection geometrical position when the X-ray shots at the assumed corresponding position. This is the reason why the aforementioned phenomena were observed when the sampling interval gets bigger. This actually states that when Katsevich's algorithm is employed for reconstruction, sampling interval between each projection data must be taken into careful consideration so as to minimize the reconstruction error as much as possible.

Visually, the reconstruction from CL scheme looks smoother than that from CH scheme; all the streak artifacts noticed in CH are gone in CL. This can also be appreciated from the profile comparison. The profile comparison in Figure 9 shows that the CL compensates density drop artifacts a little better than CH while behave the similar geometrical correction effect as CH does. This actually confirms our conjecture that by partially filling the missing Radon volume through the proposed CH, the reconstructed image quality in terms of correction to those artifacts is close to exact reconstruction; moreover, the qualitative error measurement conducted in Section 3.2.4 confirmed our conjecture. However the number of X-ray shots is quite different for these two auxiliary trajectories, 64 for CH and 556 for CL, which is a big issue considering the extra X-ray exposure level to the patient. Furthermore, the practical operation of CH is much easier than CL in which adaptively changing of X-ray collimation poses an impossible mechanical realization. Simulated quantum noise study conducted in Section 3.2.5 based on mathematic breast phantom showed that the proposed CH scheme works as good as CL scheme.

In conclusion, by incorporating a sparse partial helical scanning trajectory into an FDK-based single circular scanning scheme, a new circle plus partial helical scanning scheme was proposed to compensate for the artifacts inherited by a single circular scan for CBBCT prototype system. The numerical simulation study has demonstrated its feasibility.

## Figures and Tables

**Table 1:** Partial helical scan parameters.

Iso distance	Magnification factor	Detector pixel pitch	Number of projections	Detector size	Scanning starting position	Scanning ending position	Sampling interval along scanning axis
650 mm	1.43	0.388 mm	32	661 × 661	*Z* = 49 mm	*Z* = 121 mm	Δ*l* = 2.34 mm
			(64)				(Δ*l* = 1.15 mm)

**Table 2:** Straight line scan parameters.

Iso distance	Magnification factor	Detector pixel pitch	Number of projection	Detector size	Scanning starting position	Scanning ending position	Sampling interval along scanning axis
650 mm	1.43	0.388 mm	556	661 × 661	*Z* = 0 mm	*Z* = 510 mm	Δ*l* = 0.582 mm
			(210)				(Δ*l* = 1.552 mm)
			(64)				(Δ*l* = 5.355 mm)

**Table 3:** * RE* (%) of the numerical breast phantom among MFDK, CH, and CL.

Scan scheme	MFDK (circle)	CH (64 shots in Helix scan)	CL (556 shots in Line scan)
*RE* (%)	2.1	0.70	0.70
